# Sequencing of the whole mitochondrial genome of the Brazilian stingless bee; *Melipona scutellaris*

**DOI:** 10.1186/1753-6561-8-S4-P152

**Published:** 2014-10-01

**Authors:** Manuella Silverio, Ana Bonetti, Carlos Vieira, Cynara Rodovalho

**Affiliations:** 1 Universidade Federal de Uberlândia, Santa Mônica, Uberlândia, Brazil; 2 Instituto Oswaldo Cruz, Fiocruz, Rio de Janeiro, Brazil

## Background

In the America, from Mexico to Argentina, the genus Melipona has a great diversity of species, mainly in the Amazonian region, and a crucial pollination role. It is already well-known the importance of Brazilian stingless bees as pollinators, as well as producers of good honey, pollen, wax and propolis [1]. The present study is about the characterization of the mitochondrial genome of *Melipona scutellaris*, a Brazilian stingless bee, commonly found in the Northeast Region [2].

At first, the characterization of bees was based mostly on morphological and behavioral criteria. With advances in molecular biology, such visual criteria proved not to be reliable in all cases. Since the mitochondrial genome is well conserved among this animal species, it has been effectively used as a phylogenetic and evolutionary tool. Furthermore, the interaction between mitochondrial and nuclear genomes has important functions in the gene regulation of both [3].

Basically, the mitochondrial genome is about 16 kb long and it is composed approximately of 37 genes, of which there are the two ribosomal subunits (12S and 16S), 22 tRNAs and 13 proteins (three subunits of cytochrome oxidase, cytochrome B, subunits 6 and 8 of ATP synthase and seven subunits of NADH dehydrogenase) and still an A/T repetitive region [1]. In this study, our reference organism is the *Melipona bicolor*, a stingless bee also found in Brazil.

## Methods

### Expressed Sequence Tags (ESTs)

Previously, transcripts were obtained from the fat body of Melipona scutellaris workers bees. A cDNA library was generated and ESTs were sequenced by means of Sanger sequencing.

### DNA amplification

DNA amplification was performed using "universal" [4] and specific mitochondrial primers of Atta laevigata, a leafcutter ant. Of 15 set of primers 13 amplified, resulting in some unspecific bands. As we succeed to obtain specific bands, the PCR products were cloned using pGEM^®^-T Easy Vector (PROMEGA, US). The samples were sequenced using the MEGA-BACE 1000 DNA Sequence System (GE Healthcare, UK).

### Bioinformatics

The sequences identifications were made using Blast2Go and the NCBI database. The contigs were obtained using CAP3. Finally, they were mapped with our reference mitochondrial genome.

### Sequencing by solid

The Larvae RNA-Seq was performed using the whole RNA, except rRNA. The single end library was constructed and sequenced using SOLiD™ (Life Technologies, US). The reads were aligned to the reference mitochondrial genome using BioScope.

## Results and discussion

The sequences of PCR products and ESTs, together covered 28% of the reference mitochondrial genome. Since the mitochondrial genome is relatively short, it has been proved that long PCR products, obtained from high fidelity enzymes, are effective to cover the whole mitochondrial genome in Metazoa species [5]. Therefore, we propose next, using specific primers of M. scutellaris, designed based on the sequences already generated. Still, through SOLiD™ sequencing, we obtained reads of mitochondrial sequences. Analyzing these data, we expect to obtain a considerable part of the transcriptome, which will be completed by amplification of DNA with specific primers. The complete mitochondrial genome from M. scutellaris can provide new information about evolution in stingless bees and show new information with biotechnological potential.
